# Epithelial Cell Mitochondrial Dysfunction and PINK1 Are Induced by Transforming Growth Factor- Beta1 in Pulmonary Fibrosis

**DOI:** 10.1371/journal.pone.0121246

**Published:** 2015-03-18

**Authors:** Avignat S. Patel, Jin Woo Song, Sarah G. Chu, Kenji Mizumura, Juan C. Osorio, Ying Shi, Souheil El-Chemaly, Chun Geun Lee, Ivan O. Rosas, Jack A. Elias, Augustine M. K. Choi, Danielle Morse

**Affiliations:** 1 Division of Pulmonary and Critical Care Medicine, Brigham and Women’s Hospital and Harvard Medical School, Boston, Massachusetts, United States of America; 2 Department of Pulmonary and Critical Care Medicine, Asan Medical Center, University of Ulsan, College of Medicine, Seoul, South Korea; 3 Section of Pulmonary and Critical Care Medicine, Yale University School of Medicine, New Haven, Connecticut, United States of America; 4 Pulmonary Fibrosis Program, Lovelace Respiratory Research Institute, Albuquerque, New Mexico, United States of America; 5 Alpert Medical School, Brown University, Providence, Rhode Island, United States of America; 6 Department of Medicine, Weill Cornell Medical College, New York, New York, United States of America; Helmholtz Zentrum München, GERMANY

## Abstract

**Background:**

Epithelial cell death is a major contributor to fibrogenesis in the lung. In this study, we sought to determine the function of mitochondria and their clearance (mitophagy) in alveolar epithelial cell death and fibrosis.

**Methods:**

We studied markers of mitochondrial injury and the mitophagy marker, PTEN-induced putative kinase 1 (PINK1), in IPF lung tissues by Western blotting, transmission electron microscopy (TEM), and immunofluorescence. *In vitro* experiments were carried out in lung epithelial cells stimulated with transforming growth factor-β1 (TGF-β1). Changes in cell function were measured by Western blotting, flow cytometry and immunofluorescence. *In vivo* experiments were performed using the murine bleomycin model of lung fibrosis.

**Results:**

Evaluation of IPF lung tissue demonstrated increased PINK1 expression by Western blotting and immunofluorescence and increased numbers of damaged mitochondria by TEM. In lung epithelial cells, TGF-β1 induced mitochondrial depolarization, mitochondrial ROS, and PINK1 expression; all were abrogated by mitochondrial ROS scavenging. Finally, *Pink1* ^-/-^ mice were more susceptible than control mice to bleomycin induced lung fibrosis.

**Conclusion:**

TGF-β1 induces lung epithelial cell mitochondrial ROS and depolarization and stabilizes the key mitophagy initiating protein, PINK1. PINK1 ameliorates epithelial cell death and may be necessary to limit fibrogenesis.

## Introduction

Mitochondria play a vital metabolic role but can also cause cellular damage via the toxic byproducts of oxidative phosphorylation[1,2]. Damaged mitochondria trigger apoptosis through release of cytochrome c into the cytosol[3] and activation of cell death pathways. Cellular survival therefore depends upon efficient clearance of dysfunctional mitochondria. A selective form of autophagy, dubbed mitophagy, is a major avenue for the removal of these mitochondria. During mitophagy, mitochondria are sequestered in double-membrane vesicles and delivered to lysosomes for degradation. The pathways for generating mitophagy overlap considerably with those required for general autophagy; however, in mammals, mitochondria-specific mechanisms have been recently identified and are primarily mediated by PTEN-Induced Putative Kinase 1 (PINK1)[4] and the E3 ubiquitin ligase, Parkin[5].

PINK1 is a serine/threonine kinase that contains a mitochondrial targeting sequence[6]. In healthy mitochondria, PINK1 is constitutively imported to the inner membrane, where it is cleaved by several proteases and ultimately proteolytically degraded[7,8,9]. Loss of the mitochondrial membrane potential gradient impedes the import of PINK1 to the inner membrane, stabilizing it on the mitochondrial outer membrane where PINK1 serves as a marker of mitochondrial damage[10]. Accumulation of PINK1 on the surface of depolarized mitochondria triggers recruitment of Parkin from the cytosol. Through mechanisms that are not yet fully elucidated, Parkin stimulates mitophagy by activating components of the general autophagic machinery[11].

While the role of mitophagy in pulmonary fibrosis has not been investigated, ROS generated from mitochondria are thought to promote fibrogenesis in the lung. Most data are derived from studies of asbestosis, where H_2_O_2_ production by mitochondria of alveolar macrophages has been shown to contribute to disease pathogenesis[12]. ROS production in structural lung cells such as epithelial cells[13] is also likely to play an important role. Additionally, mitochondria-derived ROS can trigger Nalp3 inflammasome activation, which has recently been implicated in the development of lung fibrosis[14,15].

The relationship between transforming growth factor-β1 (TGF-β1) and oxidative stress is a palimpsest with multiple layers and bidirectional signaling. TGF-β1 is known to stimulate ROS production[16], and oxidative stress can activate latent TGF-β1[17,18], setting up a profibrogenic cycle. Although TGF-β1 can increase ROS production by several distinct mechanisms, the mechanism most relevant to our studies is enhanced mitochondrial ROS production via complex IV inhibition in lung cells[19]. Genetic disruption of mitochondrial complex III-generated ROS production was recently shown to attenuate TGF-β1-induced profibrotic gene expression[16], further implicating mitochondrial ROS as important players in the development of lung fibrosis.

Given the known relationship between TGF-β1 and mitochondrial ROS production, and given the known contribution of ROS to fibrogenesis, we asked whether mitochondrial clearance is altered in idiopathic pulmonary fibrosis (IPF), and whether disruption of the critical mitophagy protein, PINK1, would alter the outcomes of *in viv*o and *in vitro* models of lung fibrosis.

## Materials and Methods

### Ethics Statement

This study involves the analysis of human clinical samples. Lung tissue procurement was completed under Protocol #2012-P-001134 which was approved by the Institutional Review Board of Brigham and Women’s Hospital. Informed written consent was obtained from all study participants. All animals were housed in accordance with guidelines from the American Association for Laboratory Animal Care. Protocols were approved with Institutional Animal Use and Care Committee of Brigham & Women’s Hospital and Harvard Medical School (Protocol #04551). All experiments were carried out in a manner to minimize suffering.

### Human Subjects

All IPF subjects met diagnostic criteria of the American Thoracic Society / European Respiratory Society /Japanese Respiratory Society / Latin American Thoracic Association. Human lung tissues were procured from patients undergoing lung transplantation or donor lungs that were not implanted.

### Animal Experiments

We thank Dr. Jie Shen (Harvard Medical School/BWH) for providing the *Pink1* ^-/-^ mice[20]. *Pink1* ^-/-^ mice and their littermate controls (male, 8–10 weeks) were treated with 3 U/kg intratracheal bleomycin sulfate (RPI Corp., Mount Prospect, IL). The mice were sacrificed and lungs harvested on Day 21. Hydroxyproline assay was performed as previously described[21].

### Cell Culture

Beas-2B cells (ATCC, Manassas, VA) were cultured in DMEM (Invitrogen, Carlsbad, CA). After overnight serum starvation, cells were treated with 5 ng/mL TGF-β1 (Sigma, St. Louis, MO) for 24h unless otherwise indicated. For certain experiments, cells were co-treated with mitoTEMPO (200 μM; Enzo, Farmingdale, NY). Type II alveolar epithelial cells were isolated as previously described[21]. Carbonyl cyanide m-chlorophenyl hydrazone (CCCP) (Sigma) was used as a positive control for inducing mitophagy.

### RNA interference

PINK1 siRNA (Fisher, Pittsburgh, PA) was transfected using Lipofectamine RNAiMAX (Invitrogen) according to manufacturer’s protocol.

### Western Blotting

Protein was extracted from lung homogenate or cell lysate using RIPA buffer or Mitochondria Isolation Kit (Pierce, Rockford, IL), separated on NuPAGE 4–12% Bis-Tris gels (Invitrogen) and transferred to PVDF membranes by electroblotting. Primary antibodies used were PINK1 (Novus, Littleton, CO), β-actin (Sigma), COX-4 (Santa Cruz Biotechnology, Dallas, TX), phospho-DRP1 (Ser 616; Cell Signaling, Beverly, MA), VDAC (Cell Signaling). Secondary antibodies were HRP-conjugated goat anti-rabbit or goat anti-mouse (Santa Cruz).

### Flow cytometric analysis

Mitochondrial ROS was measured by MitoSOX (Invitrogen) staining (5 μM). For study of mitochondrial membrane potential, cells were stained with 2 μM JC-1 (Invitrogen). Apoptotic cells were detected using 10 μl/ml Annexin V (BioVision, Milpitas, CA). Data were acquired with BD FACS Canto II flow cytometer and analyzed with FlowJo (Treestar, Ashland, OR).

### Transmission Electron Microscopy and Fluorescence Microscopy

Lung tissue sections were fixed in 1.25% formaldehyde, 2.5% glutaraldehyde and 0.03% picric acid in 0.1 M Sodium cacodylate and embedded in epon. Images were obtained using a Tecnai G2 Spirit BioTWIN TEM and AMT2k CCD camera. Immunofluorescence was performed with CellLight BacMAM GFP-Mito, RFP-Lyso vectors (Life Technologies), PINK1(Novus) and LC3B(Sigma) antibodies, AlexaFluor 488(Invitrogen), Cy3(Jackson ImmunoResearch Lab., West Grove, PA) and DAPI(Invitrogen) dyes. Images were taken and processed with Olympus FluoView FV1000 confocal microscope. Image analysis of colocalization was performed with ImageJ.

### Statistical analysis

Results were expressed as mean ± SD from at least three independent experiments. Differences in measured variables between experimental and control group were assessed by using the Student’s t-test. Statistically significant difference was accepted at p<0.05.

## Results

### IPF lung tissue demonstrates increased PINK1 expression and damaged mitochondria

**Fig 1:**
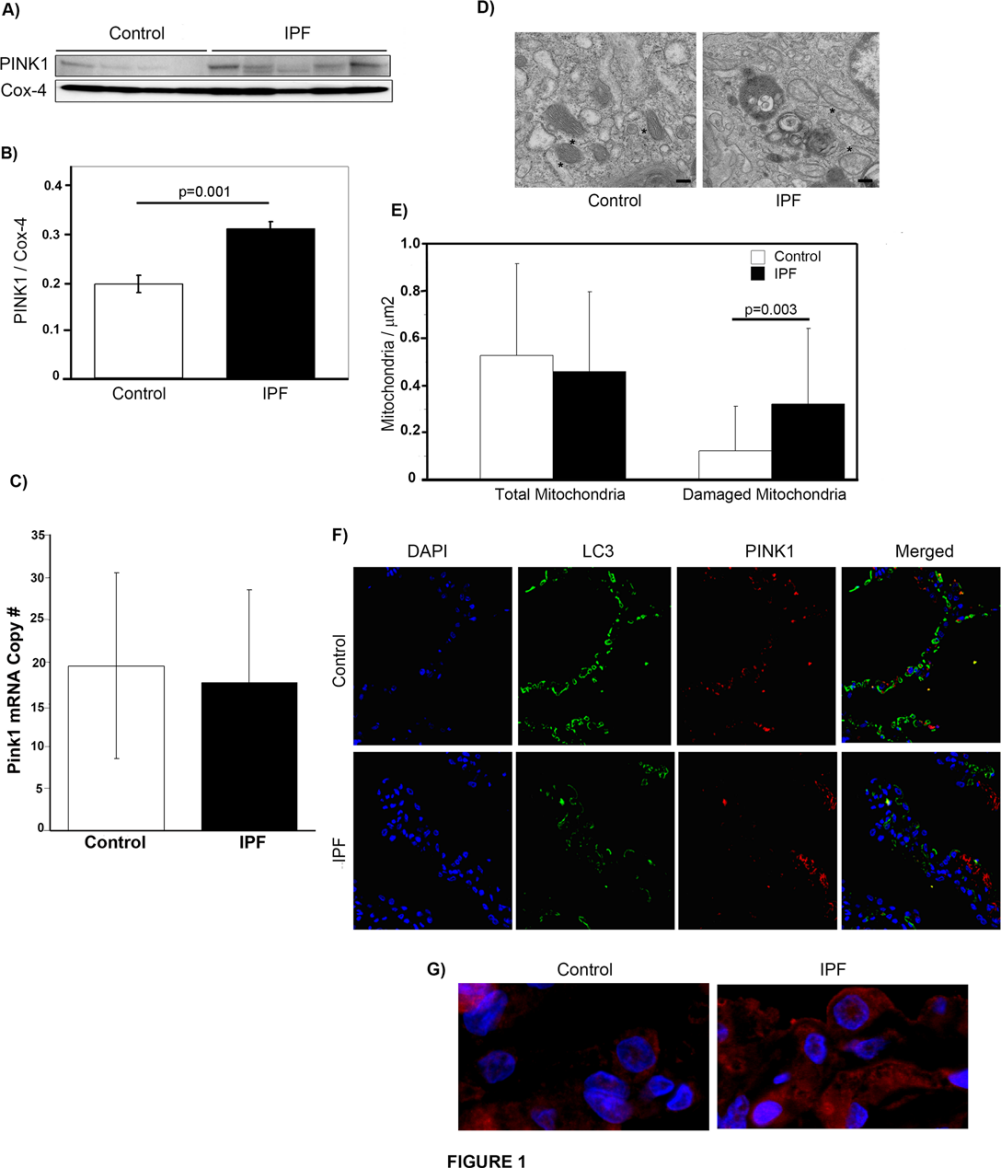
PINK1 Expression and Mitochondrial Dysfunction in IPF Lung Tissue. A) Western blot of mitochondrial fraction of PINK1 in human lung tissue from control and IPF patients showing increased PINK1 levels in IPF samples. B) Densitometry of blot in A. C) mRNA expression of Pink1 in human lung tissue from control (n = 5) and IPF (n = 5) samples. D) Representative transmission electron micrographs from control and IPF lung tissue showing mitochondria (*). Magnif = 18500x; scale bars = 200nm. E) Quantification of total and abnormal mitochondria. Total mitochondria per μm^2^ in control and IPF was 0.52 ± 0.39 vs. 0.46 ± 0.34. Abnormal mitochondria per μm^2^ in control and IPF was 0.12 ± 0.19 vs. 0.32 ± 0.32, *p* = 0.003. F) Confocal immunofluorescence against PINK1(red) and LC3 (green) in control and IPF lung (magnification 10x). G) Magnified view (60x) of confocal immunofluoresence with DAPI (blue) and PINK1 (red) in control and IPF lung.

PINK1 accumulation on the outer membrane of mitochondria is the key signal for triggering mitophagy[8]. In order to determine whether PINK1 expression is higher in lungs of IPF patients, we performed Western blotting of the mitochondrial fraction from lung tissue homogenate. As shown in Fig. 1A & B, PINK1 expression was higher in mitochondria isolated from IPF lung. In order to determine if the change in expression was due to upregulated gene expression, we performed qPCR on whole lung homogenate from control and IPF tissue (Fig. 1C). This demonstrated no change in mRNA levels, suggesting that the increased protein expression is secondary to stabilization of PINK1 in IPF. We further analyzed IPF lungs for evidence of mitochondrial dysfunction by examining mitochondrial morphology by transmission electron microscopy (Fig. 1D). A quantitative analysis (Fig. 1E) was performed by counting and analyzing mitochondria in 47 and 27 randomly selected EM fields in the IPF and control samples respectively. The number of mitochondria was normalized to area. We found a significantly increased number of mitochondria with swollen appearance or disorganized cristae in IPF lung samples when compared to control lung.

In order to better assess the cellular distribution of PINK1 in IPF and control lungs, we performed immunofluorescent staining for PINK1 in conjunction with LC3, an important component of autophagosomal membranes. In keeping with our previous findings[21], LC3 levels were higher in control lungs than IPF lungs (Fig. 1F). PINK1 expression was detected in both IPF and control lung, however, the most striking finding of our immunostaining was the difference in PINK1 cellular distribution in IPF lungs when compared with control tissue. In IPF, intense staining was seen throughout the cytoplasm of individual cells, whereas in controls, the staining was restricted to scattered cytoplasmic puncta. An example of this is shown in the high power (60x) magnification view in Fig. 1G. For this study, we did not stain with markers of various lung cell types, the populations of which may vary between healthy and diseased lung.

### TGF-β1 induces PINK1 expression in epithelial cells

Given the central role of TGF-β1 in the development of fibrosis, we examined whether exposure of cells in culture to active TGF-β1 would affect expression of PINK1. Because immunofluorescent staining of human lung (shown in Fig. 1) revealed high expression of PINK1 in epithelium, we focused on epithelial cells for our *in vitro* studies.

**Fig 2:**
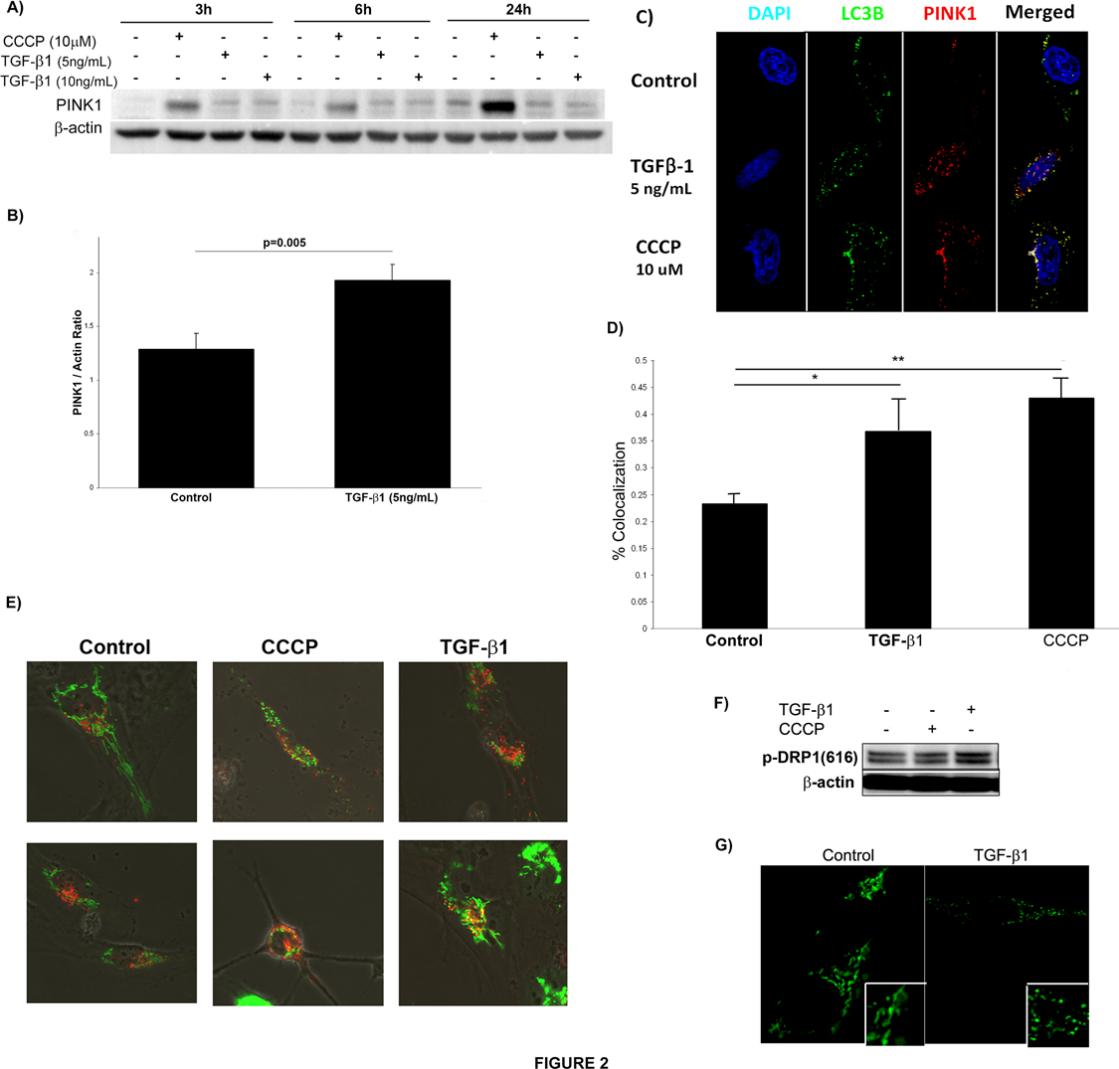
TGF-β1 increases PINK1 expression and induces mitochondrial fission *in vitro*. A) TGF-β1 induced PINK1 expression of Beas-2B cells in a time- and dose-dependent manner. B) Quantification by densitometry of PINK1 expression in Beas-2B cells stimulated with TGF-β1 for 6 hours. C) Confocal microscopy of Beas-2B cells stimulated with TGF-β1 (6h) showed that TGF-β1 induces formation of PINK1 puncta (red) (magnification 120x). D) Quantification of colocalization of LC3 and PINK1 punctae (* p = 0.035, ** p = 0.001). E) Confocal microscopy of Beas-2B cells transfected with vectors staining mitochondria (green) and lysosomes (red) and with TGF-β1 stimulation for 6 hours (magnification 60x). F) Western blot of Beas-2B cells stimulated with TGF-β1 (6hrs) showed increased expression of pDRP1 (ser616). G) Confocal microscopy of Beas-2B cells stimulated with TGF-β1 (5 ng/mL, 24 hrs) showed more fragmentation (fission) of mitochondria stained with MitoTracker Green (magnification 120x).

Fig. 2A shows PINK1 expression in Beas-2B cells treated with 5 or 10 ng/mL of TGF-β1 at timepoints up to 24 hours and demonstrates that TGF-β1 induced PINK1 expression at the early timepoints of 3 hours and 6 hours but less so at 24 hours. The mitochondrial uncoupler CCCP was used as a positive control. CCCP is a nonspecific ionophore that causes severe loss of mitochondrial membrane potential[22]. Fig. 2B shows densitometry for PINK1 expression at 6 hours by Western blot from 3 independent experiments.

If the increase in PINK1 induced by TGF-β1 correlates with enhanced mitophagy, we would expect recruitment of LC3 to the mitochondria decorated with PINK1. Fig. 2C shows that TGF-β1 does not increase LC3 levels (consistent with previous findings [21]), but co-localization of PINK1 and LC3 can be appreciated. Interestingly, there is less co-localization of PINK1 and LC3 in TGF-β1 exposed cells compared with control cells exposed to CCCP (Fig. 2D), implying that TGF-β1 may be a less efficient promoter of mitophagy than CCCP.

In order to more directly evaluate the fate of mitochondria after TGF-β1 treatment, we transfected Beas-2B cells with CellLight BacMAM vectors (Life Technologies) that labeled mitochondria with GFP and lysosomes with RFP. After transfection, cells were stimulated with TGF-β1 and analyzed by confocal microscopy at 6hrs and 24hrs (Fig. 2E). In the control cells, mitochondrial and lysosomal puncta are separate. In the CCCP treated control, there is significant association, suggesting that mitochondria are being recruited to the autolysosome. The TGF-β1 treated cells also demonstrate an association between mitochondria and lysosomes.

Although an understanding of the relationship between mitochondrial fission/fusion dynamics and mitophagy is still evolving, reduced capacity for fusion has been associated with enhanced mitophagy[23]. On the other hand, mitochondrial fission appears to be necessary for mitophagy initiation[24]. Dynamin-related protein 1 (DRP1) is a major component of the mitochondrial fission machinery in humans; when phosphorylated at Ser 616, DRP1 activity increases. TGF-β1 stimulation of Beas-2B cells resulted in enhanced phosphorylation of DRP1 (Fig. 2F) with concomitant evidence of mitochondrial fission. This can be appreciated in Fig. 2G, where cells stimulated with TGF-β1 for 24 hours demonstrated greater fragmentation of the cytosolic areas stained with MitoTracker Green. Increased mitochondrial fission in the setting of increased PINK1 is consistent with cells preparing to initiate mitophagy in response to TGF-β1 stimulation.

### TGF-β1 induces mitochondrial depolarization

**Fig 3:**
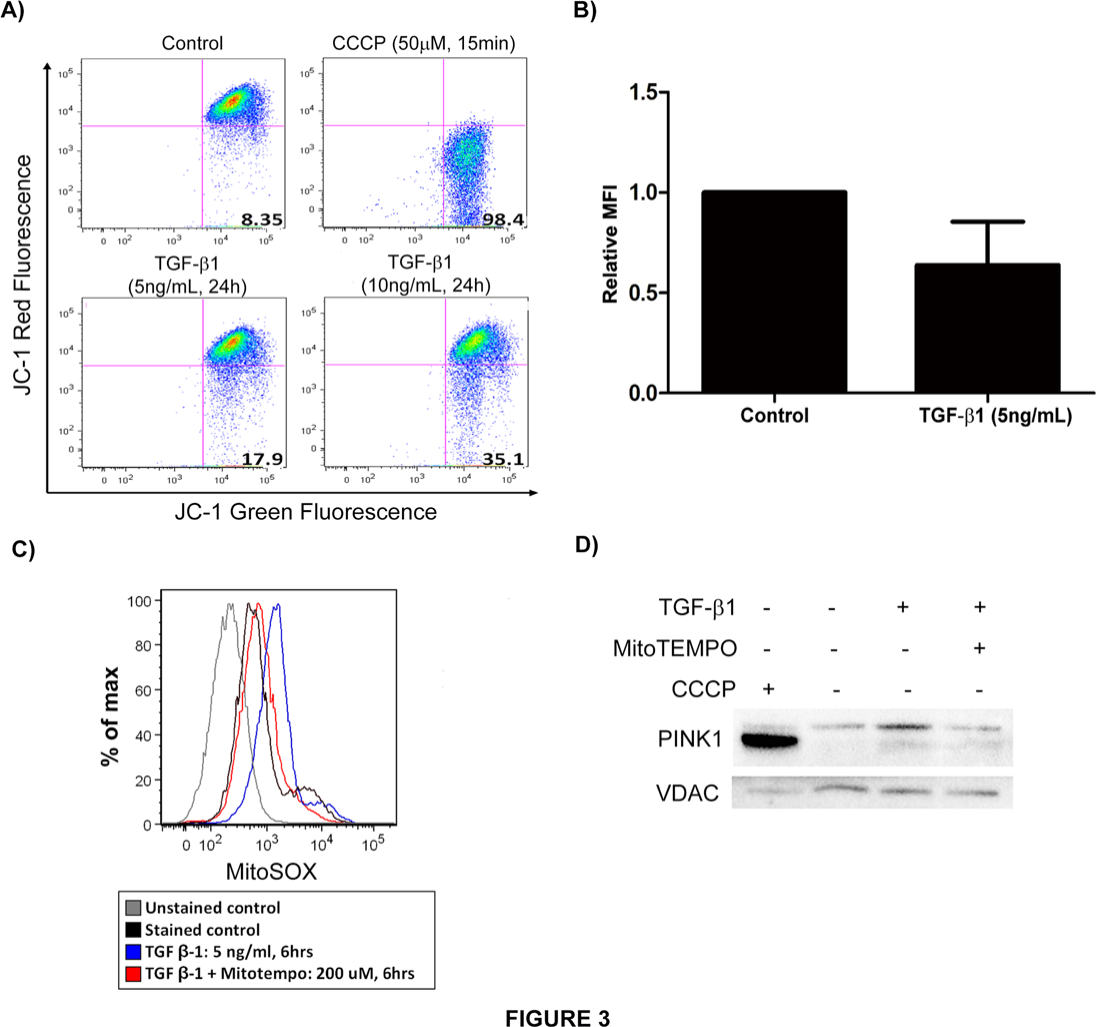
TGF-β1 induces mitochondrial depolarization. A) Beas-2B cells stimulated with TGF-β1 and CCCP (positive controls) were stained with JC-1 for 15 min and analyzed by flow cytometric analysis. TGF-β1 treated cells showed decreased red fluorescence (mitochondrial depolarization). B) Quantification of relative MFI for experiment in A (p = 0.06). C) Beas-2B cells stimulated with TGF-β1 were stained with MitoSOX for 10 min and analyzed by flow cytometry. TGF-β1 treated cells showed increased red fluorescence (mitochondrial ROS production) and mitochondria-specific antioxidant (MitoTEMPO) reversed the effect of TGF-β1. D) Western blot of the mitochondrial fraction of Beas-2B cells stimulated with TGF-β1 (5ng/mL) +/- MitoTEMPO (200μM) showing decreased PINK1 expression in presence of MitoTEMPO.

The major trigger for PINK1 stabilization on the surface of mitochondria is thought to be mitochondrial depolarization[25]. To determine whether TGF-β1 induces mitochondrial depolarization, Beas-2B cells were stimulated with TGF-β1 and subsequently stained with JC-1 (Fig. 3A and B). Flow cytometric analysis revealed the expected marked decrease in red fluorescence with CCCP treatment, the positive control, reflecting mitochondrial depolarization. In the cells treated with TGF-β1, JC-1 red fluorescence decreased in a dose-dependent manner, indicating disruption of mitochondrial membrane potential by TGF-β1.

Bursts of mitochondrial ROS associated with altered membrane potential have also been shown to induce mitophagy[26]. TGF-β1 stimulation of Beas-2B cells resulted in mitochondrial ROS production as shown in Fig. 3C. Following exposure to TGF-β1, cells were stained with MitoSOX to detect mitochondrial superoxide and subsequently analyzed by flow cytometry. TGF-β1 treated cells exhibited increased fluorescence intensity (corresponding with mitochondrial ROS production), and a mitochondria-specific antioxidant (MitoTEMPO) reversed the effect of TGF-β1, reconfirming the mitochondrial origin of the ROS. We also evaluated modulation of PINK1 expression in TGF-β1 stimulated Beas-2B cells resulting from mitochondrial ROS quenching by MitoTEMPO. At baseline, TGF-β1 induced PINK1 expression in the mitochondrial fraction, but this upregulation is abrogated in the presence of MitoTempo (Fig. 3D). We used VDAC as a mitochondrial housekeeping protein and found no effect on its expression by TGF- β1. This suggests that PINK1 induction by TGF-β1 is dependent on the ability to generate mitochondrial ROS.

### Loss of PINK1 potentiates TGF-β1 induced ROS and cell death

**Fig 4:**
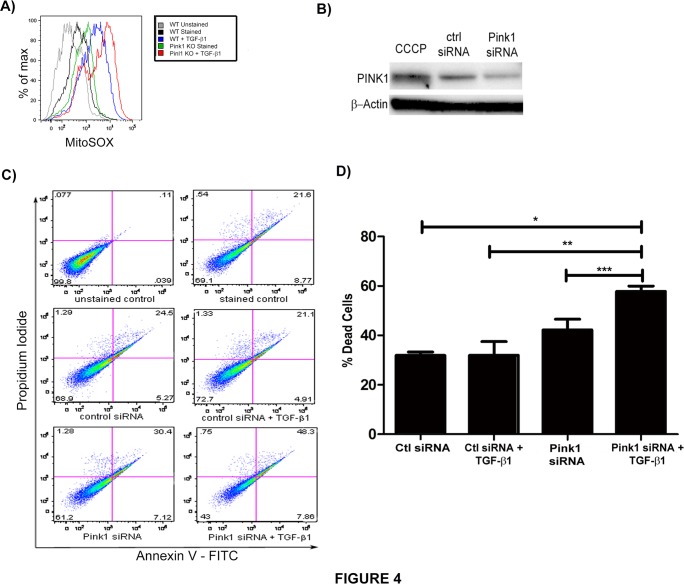
Loss of PINK1 augments TGF-β1 induced cell death. A) Measurement of mitochondrial ROS in murine type II alveolar epithelial cells from *Pink1* ^-/-^ and *Pink1* ^WT^ mice by flow cytometry and MitoSOX staining. B) Western blot against PINK1 demonstrated knockdown of expression with PINK1 siRNA. C) Beas-2B cells were treated with siRNA (50 nM, 24hrs). Then, they were stimulated with TGF-β1 (5 ng/mL, 24hrs) and analyzed by Annexin V/PI flow cytometry. D) Quantification of cell death from experiment in C (*p = 0.0005, **p = 0.0119, ***p = 0.032). Loss of PINK1 exaggerated cell death in cells treated with TGF-β1 relative to transfection with control siRNA.

TGF-β1 induced epithelial cell death is thought to be a central mechanism for the development of IPF [27]. While TGF-β1 can induce cell death through a variety of pathways[28], mitochondrial depolarization is a likely contributor to TGF-β1-induced cell death, given its prominent role in apoptotic programs. It is believed that mitophagy represents an anti-apoptotic force by removing damaged mitochondria along with their accelerated production of ROS and release of pro-apoptotic proteins[29]. To determine whether loss of PINK1 modulates mitochondrial ROS, we stimulated primary type II alveolar epithelial cells from *Pink1* ^-/-^ and *Pink1* ^WT^ mice with TGF-β1. Mitochondrial ROS were measured by MitoSOX staining using flow cytometry (Fig. 4A); this demonstrated amplified mitochondrial ROS in the absence of PINK1. To test whether inhibition of mitophagy would also enhance epithelial cell death induced by TGF-β1, we inhibited PINK1 expression in Beas-2B cells using siRNA (Fig. 4B) and then exposed the cells to TGF-β1 for 24 hours. The cells were then analyzed by flow cytometry using Annexin V and propidium iodide (PI) to quantify cell death. As shown in Fig. 4C and D, the loss of PINK1 expression resulted in exaggerated cell death in response to TGF-β1.

### Absence of PINK1 exacerbates bleomycin-induced lung fibrosis

**Fig 5:**
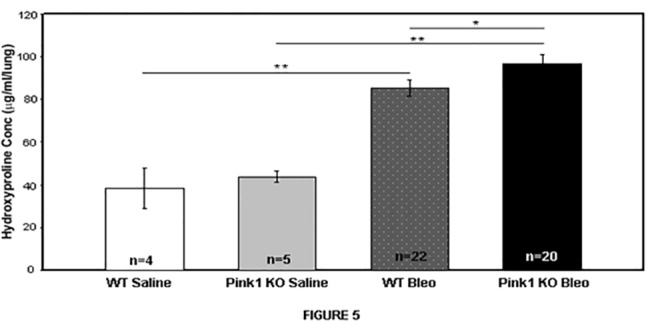
Loss of PINK1 aggravates bleomycin induced lung fibrosis. *Pink1* ^-/-^ mice and their littermate controls (male, 8–10 weeks old) were treated with 3U/kg of intratracheal bleomycin sulfate or saline on day 1 and sacrificed on Day 21. *Pink1* ^-/-^ mice in the bleomycin group showed higher levels of hydroxyproline compared to controls (85.4 μg/mL vs. 97.0 μg/mL; *p = .05, **p<0.001).

In light of the findings above, we hypothesized that upregulation of PINK1 by TGF-β1 represents an epithelial cell pro-survival response, and that inhibition of mitophagy through genetic elimination of *Pink1* would worsen experimental lung fibrosis in response to bleomycin. To test this hypothesis, we exposed *Pink1* ^-/-^ mice and littermate controls to intratracheal bleomycin or saline and harvested lungs for hydroxyproline measurement after 21 days. As shown in Fig. 5, mice with loss of PINK1 expression developed a more severe fibrotic response to bleomycin based on hydroxyproline concentrations (85.4 μg/mL vs. 97.0 μg/mL, p = 0.05), although the difference was modest at 10%. Since our hypothesis was that the absence of PINK1 would cause more epithelial cell death, we performed TUNEL staining on lung tissue sections from day 21 (data not shown). We did not find a significant difference in the number of TUNEL positive cells in the *Pink1* ^-/-^ mice compared to WT.

## Discussion

Our investigation is one of the first to address the role of PINK1, a serine/threonine kinase critical for mitochondrial clearance by autophagy, in pulmonary fibrosis. We have shown that PINK1 is more highly expressed on mitochondria from IPF lung than from control lung. Our study also demonstrates that TGF-β1 induces mitochondrial ROS and membrane depolarization, which would be expected to cause PINK1 stabilization on the mitochondrial surface, and that inhibition of mitochondria specific ROS prevents PINK1 stabilization by TGF-β1. Prevention of PINK1 upregulation by TGF-β1 enhances epithelial cell death, and genetic deletion of *Pink1* modestly exacerbates the murine pulmonary fibrotic response to bleomycin.

Our findings add to those presented in the recent publication by Bueno, et al. [30] by elucidating the role of TGF-β1 in mitochondrial dynamics. In their work, the authors found similar evidence of mitochondrial dysfunction in IPF lung tissue and type II alveolar epithelial cells (AEC) from IPF samples. Furthermore, *in vitro* experiments demonstrated decreased cell viability in the absence of PINK1. *In vivo*, *Pink1* ^-/-^ mice were more susceptible to fibrosis and were found to have increased AECII apoptosis. Interestingly, aging and ER stress were key potentiators of mitochondrial dysfunction.

The most straightforward interpretation of our findings is that TGF-β1 induces epithelial cell death at least in part through disruption of mitochondrial membrane integrity, but this effect is counterbalanced by the upregulation of PINK1 and subsequent induction of mitophagy. If mitophagy is successful in clearing dysfunctional mitochondria, then cell death is attenuated. If, on the other hand, mitophagy is inhibited via deletion of PINK1, cell death and fibrosis can proceed unchecked.

This interpretation raises a number of new questions, the most important of which is whether the machinery of mitophagy is impaired or functioning normally in the lungs of patients who develop IPF. Although we propose that mitophagy is reduced in IPF, the evidence provided is circumstantial at this point. One piece of such evidence is that autophagosome formation and LC3 activation are reduced by TGF-β1 and in IPF lung[21,31,32]. Selective and non-selective autophagy rely on common downstream machinery, and one would therefore expect mitophagy to be impacted in the same manner as non-selective autophagy in IPF. Our immunofluorescence images in Fig. 2 suggest that while TGF-β1 is a potent inducer of mitochondrial depolarization, it does not induce mitophagy *in vitro* as efficiently as our positive control (CCCP). The level of mitophagy in the fibrotic lung might therefore be insufficient to clear defective mitochondria and rescue the mitochondrial pool. This hypothesis is supported by our EM studies of IPF lung demonstrating significant numbers of damaged mitochondria. In neurodegenerative diseases, such as Parkinson’s disease and Alzheimer’s disease, studies have shown a similar accumulation of dysfunctional mitochondria leading to neuronal injury and death[33,34]. A more recent investigation in myocardial infarction has also demonstrated that attenuated mitophagy with accumulation of abnormal mitochondria promotes myocyte apoptosis and ventricular remodeling after cardiac ischemia[35]. The study by Bueno, et al. found type II AECs are similarly more susceptible to apoptosis when mitochondrial function is impaired either due to aging or induction of ER stress[30]. Thus, the paradigm of impaired mitophagy leading to increased cell death and eventual organ dysfunction may be important in the pathogenesis of multiple different diseases. The lack of definitive evaluation of mitophagy in human lung tissue is the major limitation of our work. Future studies analyzing IPF tissue for colocalization of mitochondrial and autophagy markers as well as PINK1 and mitochondrial markers could partially address this limitation and expand the understanding of PINK1-dependent and-independent mechanisms of mitophagy.

This work has several additional limitations partly stemming from the immature state of the mitophagy field. First, this study focused on the effects of TGF- β1 on PINK1 expression and PINK1 modulation of epithelial cell apoptosis but it does not shed light on other aspects of mitophagy regulation in fibrosis. It is possible that alterations in PINK1 in fibrosis are related to mitophagy independent processes, such as proteasomal stress which is also present in IPF[36,37]. Furthermore, although PINK1 is one of the few known mitophagy-specific regulators, other more relevant markers or effectors may be discovered in future. Another known effector protein is the ubiquitin ligase, Parkin. Although Parkin has not yet been studied in lung fibrosis, given its close interaction with PINK1[11], it should be the subject of future investigations. Second, we do not know to what extent the genetic deletion of *Pink1* actually inhibits mitophagy in the lung, and currently, we do not have effective tools to monitor mitophagic activity. In fact, there is evidence that mitophagy can be rescued even in the absence of PINK1[38] and that loss of PINK1 can even promote mitophagy by coordinating autophagy and mitochondrial fission machinery[39]. This may account for the modest findings in our bleomycin experiments and points to the need for other *in vivo* models, as well as assessment of specific changes in matrix protein expression and histology, to obtain a better understanding of the role of mitochondrial dysfunction and mitophagy in lung fibrosis. Furthermore, neurological studies indicate that CNS dysfunction in *Pink1* ^-/-^ mice is age dependent[40] and Bueno, et al. also demonstrate the age-dependence of mitochondrial function in lung epithelial cells[30]. Thus, the young age of *Pink1* ^-/-^ animals in our experiments may also be moderating the effect on lung fibrosis. Finally, it is certainly possible that deletion of *Pink1* has off-target effects in the lung beyond the turnover of mitochondria. Because of these limitations, the work we present here should be considered a first step in elucidating what is sure to be a complex relationship between mitophagy and lung fibrosis.

In conclusion, this study demonstrates TGF-β1 induces mitochondrial depolarization triggering PINK1 stabilization. The ability of cells to respond to this mitochondrial dysfunction is certainly a key factor in the development of pulmonary fibrosis. This study lays a foundation upon which to build a more complete understanding of mitochondrial dynamics and mitophagy in idiopathic pulmonary fibrosis.
